# G-Quadruplex binding enantiomers show chiral selective interactions with human telomere

**DOI:** 10.1093/nar/gkt1354

**Published:** 2014-01-09

**Authors:** Jiasi Wang, Yong Chen, Jinsong Ren, Chuanqi Zhao, Xiaogang Qu

**Affiliations:** ^1^Laboratory of Chemical Biology, Division of Biological Inorganic Chemistry, State Key Laboratory of Rare Earth Resource Utilization, Changchun Institute of Applied Chemistry, Changchun, Jilin 130022, China, ^2^Graduate School of the Chinese Academy of Sciences, Chinese Academy of Sciences, Changchun, Jilin 130022, China and ^3^Department of Neurosurgery, First Hospital of Jilin University, Changchun, Jilin 130021, China

## Abstract

Chiral recognition of DNA molecules is important because DNA chiral transition and its different conformations are involved in a series of important life events. Among them, polymorphic human telomere DNA has attracted great interests in recent years because of its important roles in chromosome structural integrity. In this report, we examine the short-term effect of chiral metallo-supramolecular complex enantiomers treatment on tumor cells, and find that a zinc-finger-like alpha helical chiral metallo-supramolecular complex, [Ni_2_L_3_]^4+^-P enantiomer (NiP), can selectively provoke the rapid telomere uncapping, trigger DNA damage responses at telomere and degradation of G-overhang and the delocalization of telomeric protein from telomeres. Further studies indicate that NiP can induce an acute cellular apoptosis and senescence in cancer cells rather than normal cells. These results are further evidenced by the upregulation of p21 and p16 proteins. Moreover, NiP can cause translocation of hTERT from nuclear to cytoplasm through Tyr 707 phosphorylation. While its enantiomer, [Ni_2_L_3_]^4+^-M (NiM), has no such mentioned effects, these results clearly demonstrate the compound’s chiral selectivity in cancer cells. Our work will shed light on design of chiral anticancer drugs targeting G-quadruplex DNA, and developing telomere and telomerase modulation agents.

## INTRODUCTION

DNA chiral recognition has received much attention because more and more evidences have indicated that conversions of the chirality and diverse conformations of DNA are involved in a series of important life events. Among them, polymorphic human telomere DNA has attracted great interests in recent years because of its important roles in chromosome structural integrity to protect their extremities from illegitimate recombination, degradation and end-to-end fusion (1). The human telomeres consist of long arrays of tandem TTAGGG repeats in double-stranded DNA (2–15 kb), with a G-rich single-stranded 3′-overhang of 50–400 nucleotides. During the spontaneous replicative aging, telomere is considered as a three-state model for chromosome end protection and deprotection (2). The ‘closed-state’ telomere was predicted to form a loop structure (t-loop/D-loop) for chromosome end protection (3,4). Telomere protection involves a complex of specific telomeric shelterin proteins (TRF1, TRF2, POT1, TIN2, TPP1 and Rap1) essential for genome stability (5). Deprotected telomere due to the progressive reduction of telomere length and/or damage in telomere structure activates the DNA damage response pathways, and results in a rapid cellular growth arrest and apoptosis (6–14).

Telomere maintenance is essential for cellular immortality. In most cancer cells, the integrity of telomere is maintained by a specialized reverse transcriptase named telomerase (15). Telomerase is activated in >85% of all known human tumors, but not in neighboring somatic cells (16). Therefore, interference with telomerase and telomere maintenance represents a promising strategy for anticancer therapy (17,18). Traditional strategy for direct telomerase inhibition needs a long lag period before telomeres reach the critically short length required for senescence and apoptosis to be triggered (19–21). It has also been suggested that in cancer cells, inhibition of telomerase activity might activate a recombination-based alternative lengthening of telomeres mechanism for telomere maintenance, which is one of the major limitations for the development of clinically useful telomerase inhibitors (22,23).

It has been reported that when the 3′-overhang of telomeric DNA forms quadruplex structure it cannot be elongated by telomerase (24). Therefore, small molecules that can induce and stabilize human telomeric G-quadruplex are considered as promising anticancer agents (25–29), the first example was reported by Sun *et al.* (25). This strategy can result in both shortening telomeres and directly causing telomere uncapping, which would trigger a short-term apoptosis/senescence in human cancer cells (30,31). Several types of G4 ligands have been designed to counteract telomerase and telomere for anticancer therapy (32–45). Recently we reported the first example that a chiral supramolecular complex, [Ni_2_L_3_]^4+^-P enantiomer (NiP), can selectively stabilize human telomeric G-quadruplex among different G-quadruplex and duplex DNA (46–48), and can inhibit telomerase activity (46). The complex’s preference for human telomeric DNA and its chiral selectivity can be attributed to the specific recognition of DNA loop sequence. When changing diagonal loop TTA to TTT, it eliminates NiP enantiomer chiral selectivity (47).

In this study, we examine the short-term effect of chiral metallo-supramolecular complex enantiomers treatment on tumor cells, and find that the chiral metallo-supramolecular complex, NiP, but not NiM, can induce an acute cell growth arrest and apoptosis in cancer cells rather than normal cells under the same experimental conditions. The cell growth suppression is associated with the production of DNA damage response at telomere, and provokes the rapid telomere uncapping with the degradation of G-overhang and the delocalization of TRF2, POT1 from telomere. Further studies indicate that the apoptosis is accompanied by the occurrence of β-galactosidase activity, a classical senescence phenotype. These results are further evidenced by the upregulation of p21 and p16 proteins, which have been considered as key regulators of cell cycle and cellular senescence. Upregulation of p16 and p21 can be caused by structural integrity damage rather than telomere attrition because we do not observe the reduction of telomere length under our experimental conditions. Furthermore, NiP can cause translocation of hTERT from nuclear to cytoplasm through Tyr 707 phosphorylation, while its enantiomer, NiM, has no such mentioned effects. These differences indicate that the compound has chiral selectivity in cancer cells.

## MATERIALS AND METHODS

### Preparation of metallo-supramolecular complex

The metallo-supramolecular complexes were synthesized and characterized by following literature methods (46,49,50). UV–Vis spectroscopy was used to determine the enantiomer concentration, and CD spectra of the two enantiomers prepared at the same concentration were used to estimate the purity of each enantiomer. The samples of purified M- and P-enantiomer were collected and freeze-dried, respectively, for future use (46).

### Cell proliferation assay

Cells were seeded in growth medium into T80 tissue culture flasks at 1.25 × 10^5^ cells per flask and exposed to 15 μM two enantiomers, as described above every 2–3 days and the cells were trypsinized and counted using a hematocytometer, and flasks were reseeded with 1.25 × 10^5^ cells per flask. Results were expressed as the cumulated population doubling as a function of the time of culture as described previously (51).

### Immunofluorescence

Immunofluorescence was performed as previously reported (51). Cells were fixed in 2% formaldehyde and permeabilized in 0.25% Triton X100 in phosphate buffered saline for 5 min at room temperature. For immunolabeling, cells were incubated with primary antibody and then washed in phosphate buffered saline and incubated with the fluorophore-conjugated secondary antibodies. The following primary antibodies were used: mAb anti-TRF1 (Novus), mAb anti-TRF2 (Novus), pAb anti-POT1 (Sigma), mAb anti-γ-H2AX (Genscript), mAb anti-53BP1 (Novus), mAb anti-hTERT (Rockland) and mAb anti-PCBP1 (Proteintech). The following secondary antibodies were used: Rhodamine or DyLight™488 conjugated goat anti-rabbit, fluorescein or DyLight™594 conjugated goat anti-mouse (Jackson Laboratory). Fluorescence signals were captured by using Olympus Fluoview FV1000 confocal microscope and analyzed by FV10-ASW 1.6 Viewer program (Olympus, Japan).

### Cytogenetic analysis

To determine the presence of anaphase bridges, cells were seeded on glass coverslips in complete culture medium and treated with two enantiomers for 5 days, then stained with 4′,6-diamidino-2-phenylindole (DAPI) (Sigma) and mounted. Images of anaphases were recorded with an Olympus BX-51 fluorescence microscope (Tokyo, Japan) coupled with a CCD camera controlled by DP 70 software. The frequency of anaphase bridges/micronuclei was calculated as the ratio between cells exhibiting anaphase bridges/micronuclei and the total number of anaphase cells. At least 50 anaphase cells were examined in each experiment.

Chromosome aberrations were evaluated as previously reported (51). To obtain chromosome preparations, cells in the log phase of growth were incubated with 0.1 μg/ml colchicine for 2 h and trypsinized, then incubated with hypotonic 0.075 M KCl for 10 min, fixed with methanol/acetic acid (3:1, v/v), dropped onto frosted microscope slides and air-dried overnight. Chromosomal aberrations were blindly evaluated by two independent observers in DAPI-stained metaphases from two grown cultures for each treatment. Analysis was performed at day 5 of treatment.

## RESULTS

### Chiral metallo-supramolecular complex NiP, but not NiM, induced cancer cell-specific growth suppression associated with the production of DNA damage response

**Figure 1.**
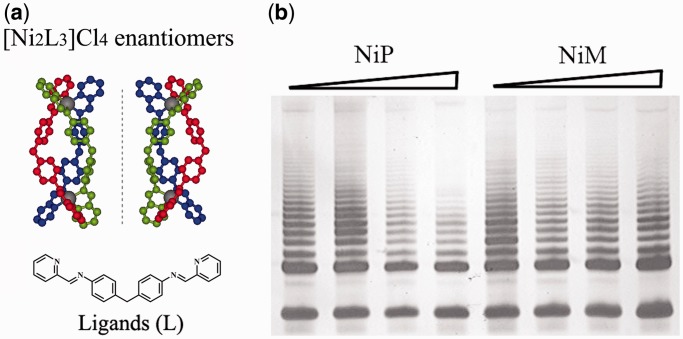
(**a**) Structures of the M-enantiomer (left) and P-enantiomer (right) of [Ni_2_L_3_]^4+^ cation. Nickel: gray; nitrogen: yellow; carbon atoms in three ligand L are shown in red, green and blue, respectively. Hydrogen atoms are omitted for clarity. (**b**) Chiral selective telomerase activity inhibition by NiM and NiP (0–15 μM) in MCF-7 cancer cells.

**Figure 2.**
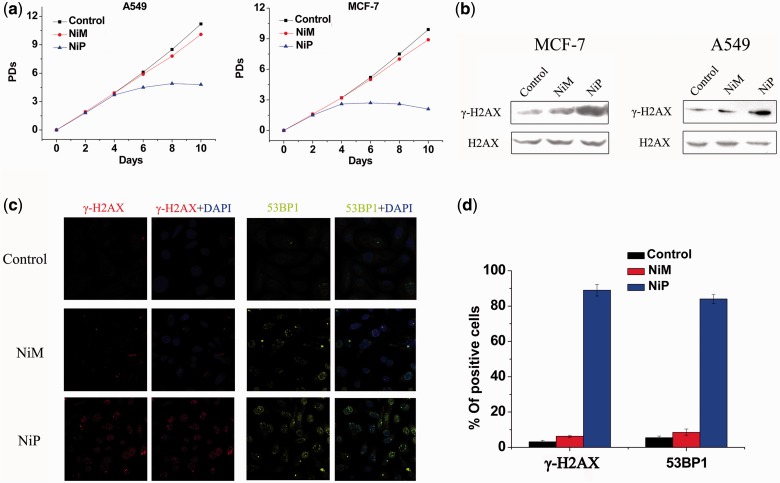
Growth suppression induced by NiP associates with the production of DNA damage response. (**a**) Proliferation curves of A549 and MCF-7 cells treated with NiM and NiP (15 μM). At the indicated times, cells were counted and the PDs were determined. (**b**) Western blot analysis of γ-H2AX in A549 and MCF-7 cells treated with NiM, NiP for 24 h. The levels of H2AX were used as loading control. (**c**) Representative immunofluorescence images of γ-H2AX and 53BP1 foci in MCF-7 cells treated with NiM and NiP. (**d**) Percentage of cells containing γ-H2AX and 53BP1 foci in MCF-7 cells treated with NiM and NiP for 24 h. γ-H2AX and 53BP1 foci were quantified using mouse mAbs. On average, >200 cells were screened in three independent experiments. Error bars indicate standard deviations (SD).

The metallo-supramolecular complexes were synthesized according to Hannon *et al.* (49,50) and characterized as previously reported (46). Experimental details were described in ‘Materials and Methods’ section. The structures of the two enantiomers of NiP and NiM were shown in Figure 1a. The telomerase activity inhibition effect of two enantiomers in human breast cancer MCF-7 cells was first investigated. As shown in Figure 1b, NiP, but not NiM, could effectively inhibit the telomerase activity in MCF-7 cells. To determine the effects of the enantiomers on cell growth at short-term treatment, we used human breast cancer MCF-7 and human lung adenocarcinoma A549 exposed to the enantiomers at a concentration of 15 μΜ, and cell viability was assessed every 2 days by Trypan blue exclusion assay. As shown in Figure 2a, for the cells treated by NiP, the decrease in cell proliferation was first observed after 3 days of treatment, followed by complete growth arrest after 1 week, which was associated with massive cell death, as shown by the apparent decrease in population doubling (PD) number between two seedings. In contrast, cells treated with the same concentration of NiM showed little effect on cell growth. To determine whether the two enantiomers were taken by cells, we analyzed for levels of Ni in cells by inductively coupled plasma mass spectrometry. As predicted, the levels of Ni were much higher in treated cells than in control (0.49 ng/10^3^ cells), and were nearly equal in cells treated by NiM (10.51 ng/10^3^ cells) and NiP (10.05 ng/10^3^ cells), indicating the chirality selectivity on cancer cells of the two enantiomers was not due to their different ability to enter the cells.

Next, we investigated whether the growth inhibition induced by NiP was associated with the production of DNA damage response. Strikingly, 24-h treatment with NiP induced a strong phosphorylation of γ-H2AX (Figure 2b), which is a hallmark of DNA double-strand break response (9,52). This result was also confirmed by the immunofluorescence for γ-H2AX and 53BP1, another DNA damage response factor (1). More γ-H2AX and 53BP1 positive cells were observed after treatment with NiP for 24 h compared with untreated groups (Figure 2c and d). However, treatment with NiM did not exhibit these effects. Notably, cell growth arrest and H2AX phosphorylation were not observed in primary culture of normal fibroblasts treated by NiP (Supplementary Figure S1), even at longer exposure time (data not shown). Based on these results, we demonstrated that growth inhibition induced by NiP was tumor cell-specific and associated with the production of DNA damage response.

### DNA damage response triggered by NiP occurred at telomere

**Figure 3.**
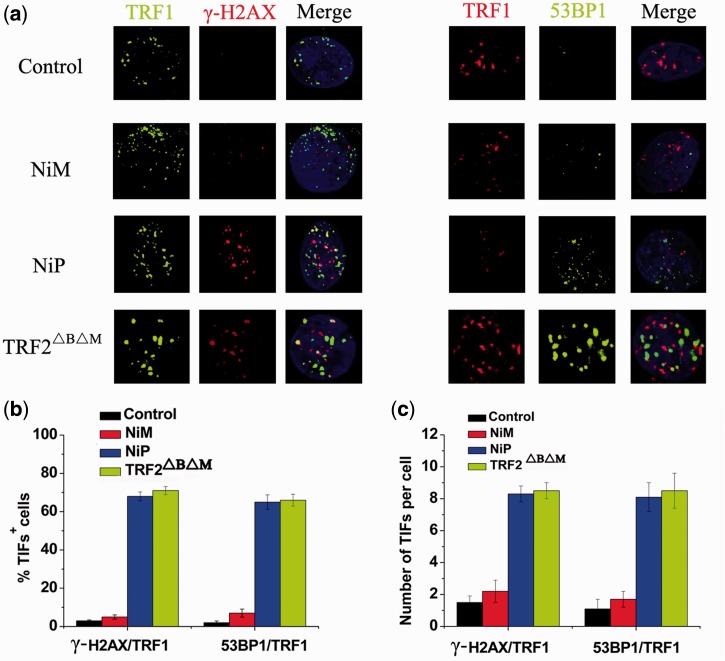
DNA damage response triggered by NiP occurred at telomeres. (**a**) MCF-7 cells expressing TRF2^ΔBΔM^ or treated with NiM or NiP for 24 h were fixed and processed for immunofluorescence using antibodies against γ-H2AX(red)/TRF1(green) or 53BP1(green)/TRF1(red), respectively. Representative confocal images were shown. (**b**) TIF index, defined as foci of DNA damage response factors that coincided with TRF1, was calculated as the percentage of TIF-positive cells in MCF-7 cells expressing TRF2^ΔBΔM^ or treated with NiM or NiP. Cells with four or more γ-H2AX/TRF1or 53BP1/TRF1 foci were scored as TIF positive. The mean of three independent experiments was reported. Error bars indicate SD. (**c**) Average number of TIFs per nucleus in MCF-7 cells expressing TRF2^ΔBΔM^ or treated with NiM or NiP. The mean of three independent experiments with comparable results was shown. Error bars indicated SD.

To verify whether γ-H2AX was activated at telomeres (53,54), double immunofluorescence experiments were performed in MCF-7 cells treated by the two enantiomers. Confocal microscopy revealed that most of the γ-H2AX foci induced by NiP colocalized with TRF1 (Figure 3a), forming the so-called telomere dysfunction-induced foci (TIFs) (9). Quantitative analysis revealed that treatment with NiP significantly increased the percentage of cells with more than four γ-H2AX/TRF1 colocalizations (the percentage of TIFs-positive cells reached ∼68% on treatment with 15 μM concentration), with a mean of ca. 8 TIFs per nucleus (Figure 3b). Telomere dysfunction induced by NiP appeared to be potent, since the percentage of cells with TIFs-positive nearly matched that measured in cells overexpressing the TRF2^ΔBΔM^ allele (9). Another DNA damage response factor, 53PB1, was also recruited to the telomeres on NiP exposure (Figure 3b), confirming that NiP triggered a genuine DNA damage response at telomeres. However, no or slight telomeric DNA damage response was found in NiM-treated cells. These results demonstrated that NiP triggered DNA damage response specifically occurred at telomeric regions.

### Evidence of telomere uncapping induced by NiP

A current model proposes that telomere forms ‘a cap’ at the end of chromosomes (1). It has been hypothesized that induction of quadruplex formation at the telomere may result in alterations of telomere capping, evidenced by the formation of anaphase bridges, micronuclei and fused telomere (36,54,55).

**Figure 4.**
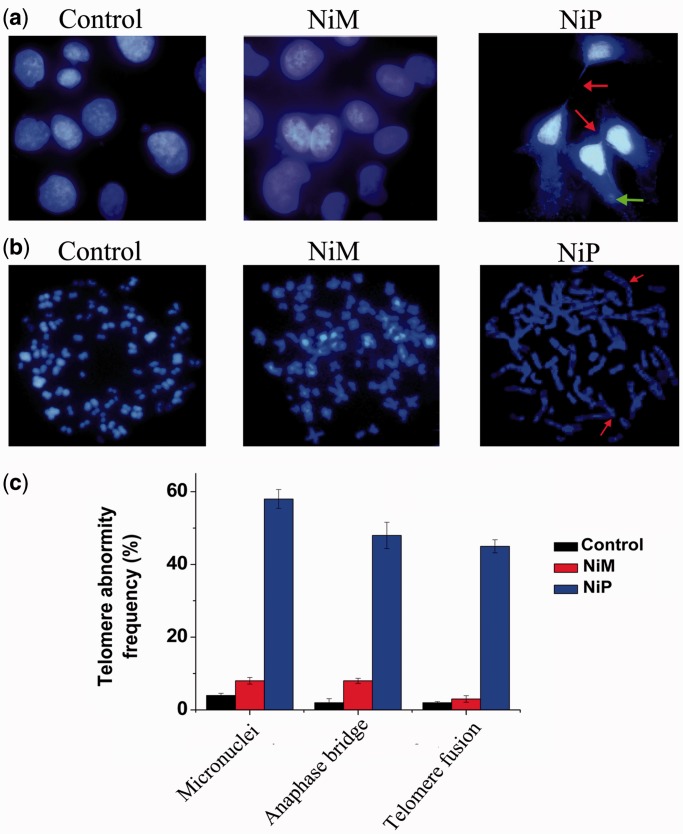
Telomere uncapping induced by NiP. (**a**) Representative images of anaphase bridges and micronuclei in MCF-7 cells treated for 5 days with NiM or NiP were shown. Cells were stained with DAPI and images were recorded (Original magnification: ×100 objective). *Red arrow* indicated bridge formation, and Green arrow indicated micronuclei formation. Anaphase bridges and micronuclei often coexisted in treated cells. (**b**) Telomere fusion induced by NiP. Metaphase spreads were stained with DAPI. *Red arrow* indicated telomere fusion. (**c**) The frequency of telomere instability was calculated as the ratio between cells exhibiting anaphase bridges/micronuclei and the total number of anaphase cells (at least 50 anaphase cells were examined). Telomeric fusion frequency was calculated as total number of telomeric fusions/total number of metaphases. The data represented the means of four independent experiments with SD.

Next we explored whether G-quadruplex stabilization induced by NiP could interfere with telomere integrity and induce formation of anaphase bridges. Telomere status was analyzed in MCF-7 cells by staining of nuclei with DAPI, performed on day 5 of treatment, and revealed that cells treated with NiP displayed typical images of anaphase bridges and micronuclei, which indicated telomere uncapping (Figure 4a). Furthermore, metaphase spreads in the treated groups were also prepared and stained with DAPI. As shown in Figure 4b and c, remarked telomere fusion was observed in NiP-treated cells, indicating the chromosome abnormality. However, treatment with NiM did not induce these multiple cytogenetic aberrations. Moreover, to directly label the unprotected telomere, a terminal deoxytransferase (TdT) assay that adds CY3-conjugated deoxyuridine to naked telomere ends was applied. In NiP-treated cells, ∼65% of TdT signals colocalized with telomeres, indicative of robust telomere uncapping (Supplementary Figure S2). However, the TdT-CY3 assay did not detect specific nuclear staining in untreated or NiM-treated cells. Taken together, these results demonstrated that NiP could induce telomere uncapping and expose chromosomal termini to the DNA damage pathway.

### NiP-induced dissociation of telomere-binding proteins from telomere

**Figure 5.**
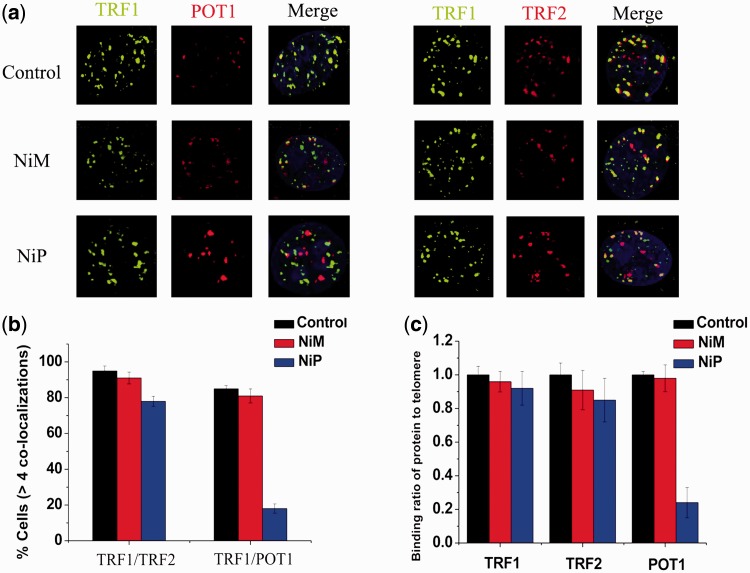
NiP specifically delocalizes TRF2, POT1 from telomeres. (**a**) MCF-7 cells treated with NiM or NiP were double stained with the indicated antibodies. Representative confocal images showing merged TRF1 (*green*) with TRF2 or POT1 (*red*) staining in untreated and treated cells. (**b**) Percentages of cells with more than four colocalizations per nucleus of TRF1/TRF2 or TRF1/POT1. Error bars indicated SD. (**c**) Binding of TRF1, TRF2 and POT1 was examined by ChIP assay and detected by qRT-PCR amplification of the telomeric region in MCF-7 cells treated with NiM or NiP. Data represented triplicate ChIP experiments, each with technical triplicates of qRT-PCR.

The telomere uncapping was usually associated with the dissociation of telomere-binding protein from telomere (53–55). We next investigated the effect of NiP on the localization of TRF2 and POT1, two telomeric proteins inducing telomere dysfunction, and evoking DNA damage signaling when their levels are reduced at telomeres (13,56–58). Confocal microscopy showed that NiP specifically delocalized TRF2, POT1 from TRF1 foci in MCF-7 cells after 2 days of treatment (Figure 5a). Quantitative analysis revealed that the percentage of nuclei with >4 TRF2/TRF1, POT1/TRF1 colocalizations was markedly reduced in cells exposed to NiP (Figure 5b). To confirm the results of these immunofluorescence analyses, we performed quantitative real time-polymerase chain reaction (qRT-PCR)-based ChIP assay as described above using the same antibodies used in the immunofluorescence experiments. As expected, NiP significantly reduced the binding of TRF2, POT1 to the telomere, without affecting the association of TRF1 to the telomere, in agreement with the immunofluorescence results (Figure 5c). We also provided evidences that the removal of TRF2, POT1 from telomere was not associated with the expression change (Supplementary Figure S3).

**Figure 6.**
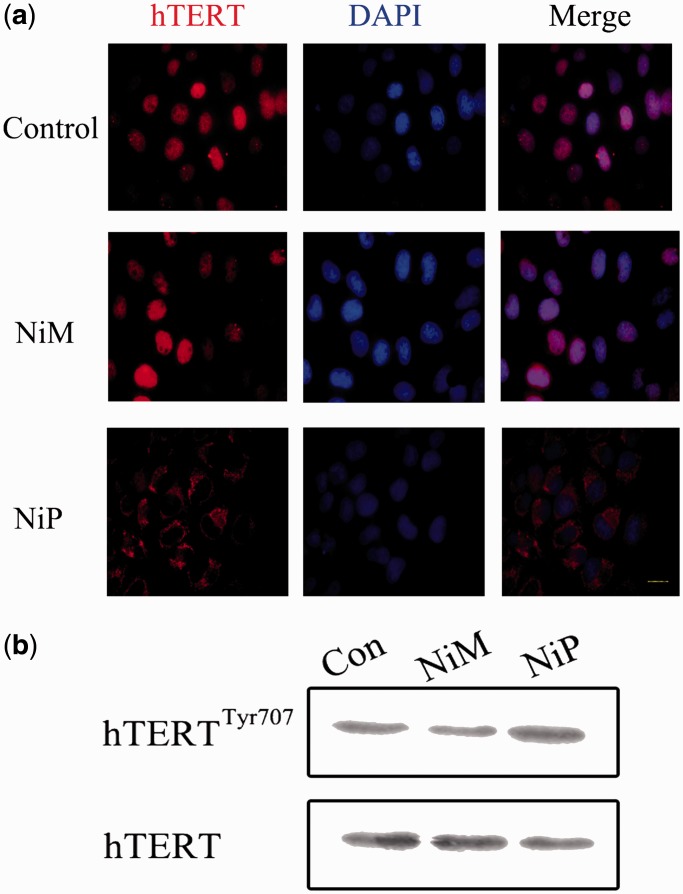
NiP treatment leads to a decrease of hTERT expression in the nucleus and translocation to cytoplasm. (**a**) MCF-7 cells treated with NiM or NiP were stained with the hTERT antibodies for hTERT and DAPI for nucleus. Representative confocal images were shown. (**b**) Western blot analysis of hTERT phosphorylation in cells exposure to NiP. The levels of hTERT were used as loading control.

The translocation of hTERT from nuclear to cytoplasm was also observed. MCF-7 cells were treated with NiP for 2 days and expression of hTERT was investigated by immunofluorescence (red). MCF-7 control cells expressed nuclear hTERT, treatment of MCF-7 cells with NiP for 2 days led to an overall reduction of hTERT protein, and in particular in nuclear localization; however, detectable hTERT levels were observed in the cytoplasm (Figure 6a). It has been established that hTERT shuttling between subcellular compartments involved in telomerase activity regulation (59–63). Although the molecular mechanism regulating nuclear localization of hTERT is unclear, the Tyr 707 phosphorylation is reported to regulate the subcellular location of hTERT (63). We found that the Tyr 707 was phosphorylated on exposure to NiP, which may charge for the translocation of hTERT under this situation (Figure 6b).

Furthermore, we investigated the effect of NiP on the telomeric G-overhang length and the total telomere length by using hybridization protection assay (HPA) (55,64). As shown in Supplementary Figure S4, NiP significantly reduced the telomeric G-overhang length after 5 days of treatment, while it did not change the total telomere length. However, cells treated with NiM did not show these effects except the slight reduction of telomeric G-overhang length. The displacement of telomere binding proteins from telomere and the G-overhang reduction induced by NiP may indicate G-quadruplex formation, as described for some other G-quadruplex ligands (53–55).

### Short-term apoptosis and senescence evoked by NiP-induced telomere dysfunction

**Figure 7.**
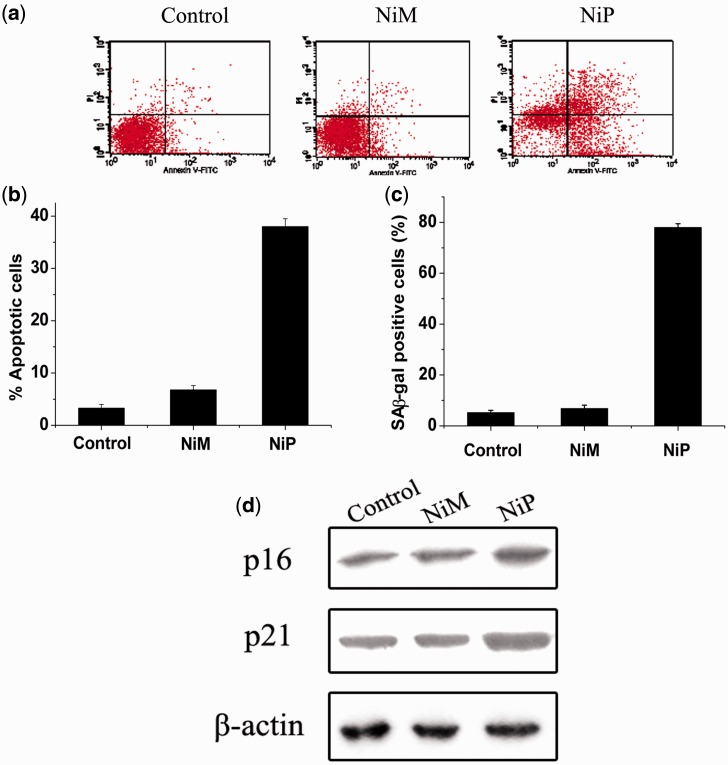
Apoptosis and senescence evoked by NiP-induced telomere dysfunction. (**a**) Apoptotic cell death induced by NiP in MCF-7 cells. After treatment with NiP, cells were collected and stained with PI and Annexin V-FITC. Representative data of flow cytometry was shown. (**b**) Annexin V positive/PI negative cells were measured by flow cytometry. The percentages of cells undergoing apoptosis were expressed with respect to the total number of cells. (**c**) Expression of senescence-associated β-galactosidase (SA-β-gal) in MCF-7 cells after continuous treatment with NiM or NiP. This assay was performed in triplicate. The senescent cells were counted under an inverted microscope in five random fields. (**d**) Upregulation of p16 and p21 proteins induced by NiP. After MCF-7 cells were treated with NiM or NiP, cells were lysed and separated on 10% SDS-PAGE and probed with anti-p16^INK4a^ and anti-p21^WAF1^ primary antibody, respectively. Immunoblotting for β-actin was also performed to verify equivalent protein loading. Each experiment has been repeated three times.

Furthermore, we explored whether telomere dysfunction induced by NiP could result in apoptosis or senescence in MCF-7 cells. Annexin V assay was performed to assess apoptosis after treatment with NiP for 5 days. As shown in Figure 7a, apoptosis occurred after incubation with NiP, but not NiM. Moreover, the apoptosis was accompanied by the occurrence of β-galactosidase activity, a classical senescence phenotype. These results were further evidenced by the upregulation of p21 and p16 proteins (Figure 7b), which have been considered as key regulators of cell cycle and cellular senescence (10,65). It should be noted that the upregulation of p16 and p21 could be caused by structural integrity damage rather than telomere attrition because we did not observe the reduction of telomere length under our experimental conditions.

Similar results were obtained in human lung adenocarcinoma A549 cells (data not shown). However, we did not observe any apoptosis/senescence in NiP-treated normal human umbilical vein endothelial cells (Supplementary Figure S5). The induction of apoptosis and the accelerated senescence have been described as one of the characteristics of G-quadruplex binding ligands in cancer cells. Based on these results, the chiral supramolecular complex NiP can not only inhibit telomerase, but also trigger a series of telomere-related cellular events and possesses chiral selectivity against cancer cells.

### Overexpression of POT1 increases G-overhang and protects cells from NiP effects

**Figure 8.**
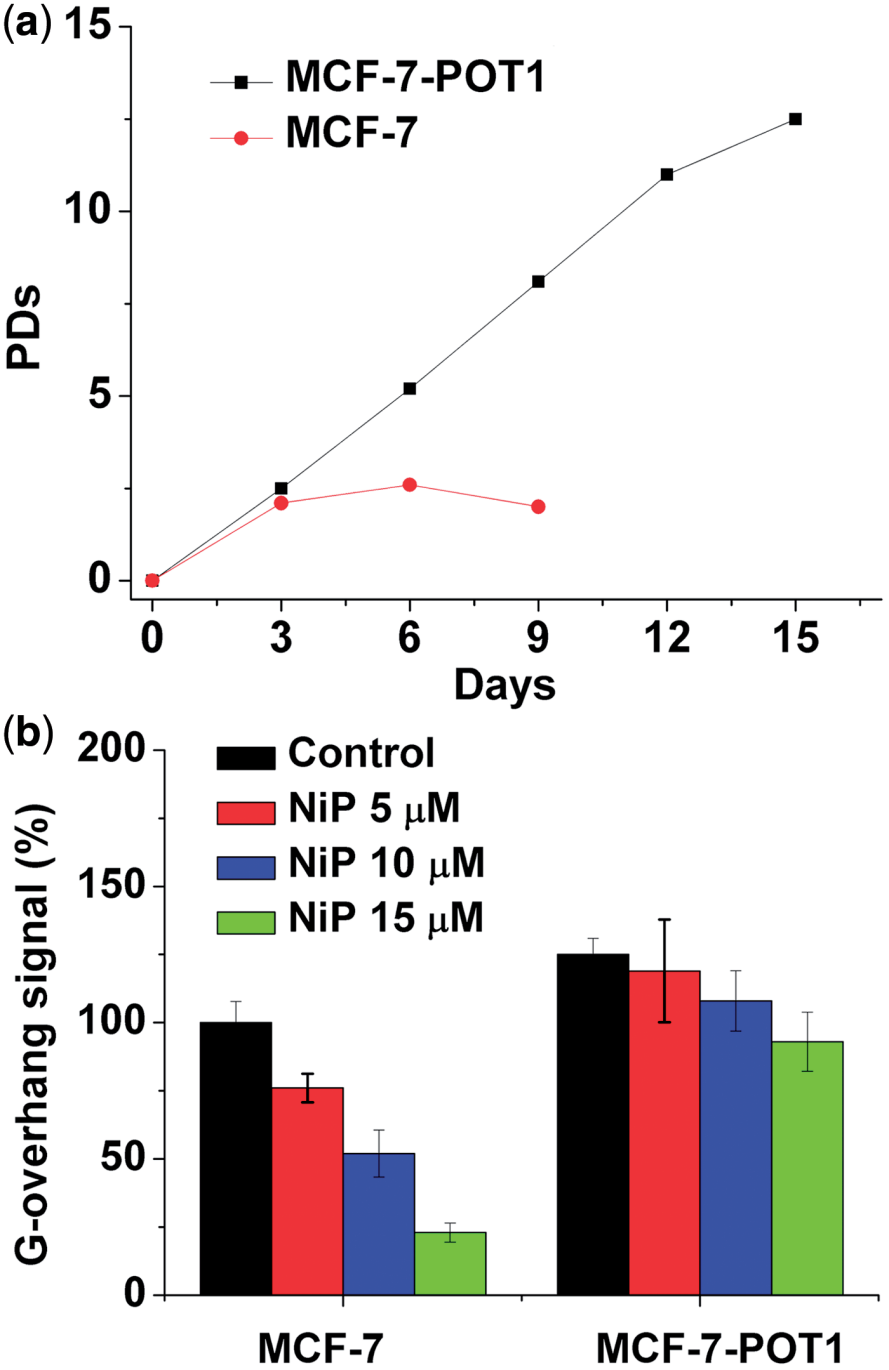
Overexpression of POT1 increases G-overhang and protects cells from NiP effects. (**a**) Proliferation curves of MCF-7 and MCF-7-POT1 cells treated with NiP (15 μM). At the indicated times, cells were counted and the PDs were determined. (**b**) Hybridization protection assay (HPA) was performed on genomic DNA isolated from MCF-7 and MCF-7-POT1 cells treated with various concentrations of NiP to assess the length of G overhang. Luminescence intensity in arbitrary units (AU) was normalized against *Alu* probe. The mean of three independent experiments with comparable results was shown. Error bars indicated SD.

POT1 protein is essential for telomere capping and allows us to regulate potential G-quadruplex structures formed at the telomeric G-overhang *in vitro* (13,56,66,67). Overexpression of POT1 may protect or modulate the telomere dysfunction induced by G-quadruplex ligands. We therefore examined whether overexpression of POT1 could modulate the cellular effects of NiP. Treatment of MCF-7 cells with NiP induced a cell growth arrest after four population doublings, followed by cell death at day 6 (Figure 8a). Interestingly, MCF-7-POT1 cells presented a noticeable resistance to the effect of NiP because the growth arrest was not observed after 15 days (Figure 8a). As a control, doxorubicin treatment of the cell lines did not indicate significant resistance in MCF-7-POT1 cells, as compared with MCF-7 (data not shown). These results indicated that reinforcement of telomere capping functions by POT1 counteracted the effects of NiP on telomeres and tumor cells.

To determine whether this protection corresponded to a difference in the effect of NiP at telomeres, we then analyzed the G-overhang degradation in these two cell lines, MCF-7 and MCF-7-POT1. We noticed that the G-overhang signal measured in MCF-7-POT1 was much higher, as compared with MCF-7 cells, indicating that the overexpression of POT1 had a positive regulatory effect on the G-overhang length (Figure 8b). Treatment of MCF-7-POT1 and MCF-7 cells with NiP for 5 days induced a dose-dependent reduction of the G-overhang signal. However, the decrease of the G-overhang signal in MCF-7-POT1 cells was lower than that in the MCF-7 cells, and the remaining G-overhang signal was therefore higher than that in treated MCF-7 cells. These results indicated that the POT1 expression protecting G-overhang from degradation played an important role in the resistance mechanism to NiP for MCF-7 POT1 cells. These results also indicated that NiP could target the telomere architecture and damage the G-overhang structure through stabilization of G-quadruplex (66,67).

## DISCUSSION

Chirality plays a pivotal role in biological reactions and the evolution of life itself. Chiral recognition is important for drug design because many drugs are chiral and usually only one of the enantiomers is pharmaceutically active, while the other may exert severe side effects. Supramolecular complexes have shown advantages for designing DNA chiral binding agents (49,68,69). In this report, we found that one of chiral supramolecular enantiomers, NiP, triggered a short-term apoptosis/senescence in MCF-7 and A549 cancer cells under our experimental conditions. This effect did not result from telomere shortening, but with rapidly telomere uncapping and G-overhang degradation, indicating that the effects induced by NiP were depended on telomere structure rather than telomere length. This hypothesis was further supported by the rapid induction of γ-H2AX foci at telomere in NiP-treated cells, which was induced by the telomere dysfunction. The percentage of cells treated by NiP with TIFs-positive nearly matched that measured in cells overexpressing the TRF2^ΔBΔM^ allele, demonstrating that NiP was an effective telomere targeting agent.

In NiP-treated cells, a rapid removal of TRF2, POT1 from telomere was found. The current model suggests a key role for TRF2, POT1 in the formation of the t-loop/D-loop structure, which protects the chromosome end from the ATM or ATR kinase pathway (1). Therefore, our results implied that the NiP targeted the very end of telomere and disrupted the t-loop/D-loop structure through the stabilization of G-quadruplex. This hypothesis was supported by the G-overhang degradation in NiP-treated cells, however, without telomere attrition. More importantly, the cells overexpressing POT1 with a longer G-overhang became resistant to NiP. POT1 overexpression might counteract the deleterious effects of NiP through masking the 3′overhang against NiP binding. However, we did not observe the obvious dissociation of TRF1 from telomere in cells exposed to NiP. The difference between TRF1 and TRF2 could be due to their different binding sites on telomere. TRF1 has a propensity for binding long tracts of double-stranded DNA and has been postulated to modulate the length of telomere via its interaction with other telomere associated proteins. In contrast, TRF2 can directly bind not only the telomeric double-stranded DNA but also the double-/single-stranded DNA junction near the 3′-overhang, and promote the overhang to invade the upstream duplex region to form the t-loop structure (1). Moreover, TRF1 has been reported to have an approximately four times higher binding affinity to telomeric DNA than TRF2 (70). Therefore, when the telomeric DNA forms quadruplex structure, TRF2 would be more sensitive and easy to dissociate from telomere than TRF1.

We also found that NiP induced the loss of the telomerase catalytic subunit hTERT (35,71). This effect could be explained owing to G-quadruplex ligand targeting telomere and causes displacement of the hTERT from the telomere (35,71), and translocation from nuclear to cytoplasm. Furthermore, our studies indicated that the Tyr 707 of hTERT was phosphorylated after the exposure to NiP. Tyr 707 phosphorylation has been reported to charge for the translocation of hTERT, demonstrating that the kinase pathway was involved in this scenario (63).

It should be noted that the normal fibroblasts were much less sensitive to NiP treatment than the cancer cells. This may be due to the longer telomere length of the normal cells. Previous studies have demonstrated that telomere length strongly influences the anticancer effect of G-quadruplex ligands (53,55). The results indicated that the G-overhang degradation played an important role in the NiP’s anticancer activity. In view of the 3′overhang length associated with the size of telomere (72,73), the 3′overhang length can be an important factor for G-quadruplex ligands against cancer cells.

Interestingly, the metallo-supramolecular complex NiM, the chiral enantiomer of NiP, could not induce short-term apoptosis and senescence associated with telomere damage under the same experimental conditions. Our previous studies have shown that the P-enantiomer selectively stabilizes human telomeric G-quadruplex over duplex DNA (46,47). Moreover, the NiP can discriminate different quadruplexes with preference to human telomeric G-quadruplex, and induce single-stranded human telomeric oligomer DNA to form G-quadruplex (48). In combination with our results reported here, NiP can target telomere and cause a series of DNA damage and cell senescence, specifically occurred in cancer cells. Telomeric G-quadruplex is an appealing drug target for cancer therapy. Although many ligands have been reported to stabilize G-quadruplexes, few examples show high selectivity towards human telomeric G-quadruplex DNA. G-quadruplex DNA is polymorphic, and its chirality transitions are involved in a series of important life events. Therefore, targeting human telomeric G-quadruplex by chiral complexes may pave a new way for developing chiral anticancer drug agents. In this report, we demonstrated the two enantiomers targeted the very end of the telomere structure with chiral selectivity, and therefore showed different anticancer activity.

In summary, we report that a zinc-finger-like chiral metallo-supramolecular complex NiP, but not NiM, induces an acute cell growth arrest and apoptosis in cancer cells. Intriguingly, NiP does not affect normal fibroblasts within 3 weeks. The short-term effect of NiP is associated with the rapid telomere uncapping with the degradation of G-overhang and the dissociation of telomere-binding protein, TRF2 and POT1, from telomere. These effects result in series of DNA damage, cell senescence, upregulation of two key regulators of cell cycle and cellular senescence, p16 and p21, and cause the translocation of hTERT from nuclear to cytoplasm through Tyr 707 phosphorylation. These results indicate that NiP can target the very end of telomere, and shows chiral selectivity in cancer cells. Therefore, our work demonstrates that chiral recognition of polymorphic human telomeric DNA is promising for design of chiral anticancer drugs, and for developing telomere and telomerase modulation agents.

## SUPPLEMENTARY DATA

Supplementary Data are available at NAR Online.

## FUNDING

973 Project [2011CB936004, 2012CB720602], and NSFC [21210002, 91213302]. Funding for open access charge: 973 Project NSFC.

*Conflict of interest statement*. None declared.

## Supplementary Material

Supplementary Data
