# Supplementary material for: G-Quadruplex binding enantiomers show chiral selective interactions with human telomere
Source: Nucleic Acids Res. 2014 Jan 9;42(6):3792–802. doi: 10.1093/nar/gkt1354 (PMC3973297; doi:10.1093/nar/gkt1354)
Supplement: Supplementary Data [file supp_42_6_3792__index.html]

G-Quadruplex binding enantiomers show chiral selective interactions with human telomere — G-Quadruplex binding enantiomers show chiral selective interactions with human telomere — Supplementary Data 

# G-Quadruplex binding enantiomers show chiral selective interactions with human telomere

## Supplementary Data

files

**Files in this Data Supplement:**

- Supplementary Data - pdf file
