# Supplementary material for: G-Quadruplex binding enantiomers show chiral selective interactions with human telomere
Source: Nucleic Acids Res. 2014 Jan 9;42(6):3792–802. doi: 10.1093/nar/gkt1354 (PMC3973297; doi:10.1093/nar/gkt1354)
Supplement: Supplementary Data [file supp_gkt1354_nar-03317-f-2013-File010.pdf]

Supporting Information

SI Materials and Methods

Cell culture and agents

Human breast cancer MCF-7, human lung adenocarcinoma A549 and human umbilical vein endothelial cells (HUVECs) were grown in Iscove's modified Dulbecco's medium (Gibco BRL) supplemented with 10% fetal calf serum in a humidified 37℃ incubator with 5% CO_2. Exponentially growing cells were used for the experiments.

Construction and Transfection of Recombinant Expression Plasmids

The *pcDNA3.1/myc- TRF2*$^{\triangle B\triangle M}$, *pcDNA3.1-POT1* mammalian expression vector were cloned as described (1). The plasmids were transfected with Lipofectamine 2000 (Invitrogen) following the manufacture′s instruction. The myc tag was detected by Western blotting.

ChIP assays

ChIP assays were performed according to manufacturer's instructions (Millipore). Briefly, Cells were fixed in 0.8% paraformaldehyde in 1× PBS, washed extensively in 1× PBS, lysed in ice-cold Lysis buffer (1% SDS, 10 mM EDTA, 50 mM Tris-HCl at pH 8.0 and protease inhibitors; sonicated chromatin products of ～100-300 bp), and diluted 10× in dilution buffer (20 mM Tris-HCl at pH 8.0, 150 mM NaCl, 2 mM EDTA, protease inhibitors, and 1 mg/mL bovine serum albumin). ChIP was performed with the relevant antibody and captured with Protein A/G–Sepharose. DNA-protein complex was washed with 1×wash buffer I (20 mM Tris-HCl at pH 8.0,

150 mM NaCl, 0.1% SDS, 1% Triton X-100, and 2 mM EDTA), 2×wash buffer II (20 mM Tris-HCl at pH 8.0, 250 mM NaCl, 0.1% SDS, 1% Triton X-100, and 2mM EDTA) and eluted in 1% SDS and 100 mM $NaHCO_3$. Eluate fraction was de-cross-linked by high-salt treatment (200 mM NaCl) at 60 ℃ followed by proteinase K treatment at 50 ℃. DNA extracted was subjected to PCR (see below).

Standard and telomere PCR analysis

Telomeric sequences in immunoprecipitates were evidenced by PCR amplification according to a method described previously (1). The final telomere primer concentrations were 270 nM (tel1) and 900 nM (tel2), and PCR amplification was subjected to 35 cycles of 95 ℃ for 15 s, 54 ℃ for 2min. The primer sequences were as following: tel1 5′-GGTTTTTGAGGGTGAGGGTGAGGGTGAGGGTGAGGGT-3′ and tel2 5′-TCCCGACTATCCCTATCCCTATCCCTATCCCTATCCCTA-3′. Quantitative PCR was done using the SYBR green Jumpstart Taq ReadyMix (TaKaRa) on a Roche LightCycler 480.

Quantification of telomere length and telomere 3′single-stranded overhang by the G-tail telomere hybridization protection assay (Gt-telomere HPA)

For HPA (2), 1 μg non-denatured genomic DNA and 0.5 μg heat-denatured genomic DNA was used per assay for the detection of telomere 3’ overhangs and total telomere DNA, respectively. Briefly, 100μL of 3 × 10^6 rlu (relative light units) of acridiniumester (AE)-labeled telomere HPA probe (5'-CCCTAA CCC TAA CCC TAA CCC TAA CCCTA-3’; AE position is underlined) in hybridization buffer was added to the DNA solution, mixed well by vortex, and incubated at 60 ℃ for 20 min.

Specific activity of AE-labeled probe was 8×10^7 rlu/pmol probe DNA. Hydrolysis of the AE of unhybridized probes was carried out by adding 300 mL of the hydrolysis buffer to each reaction tube, mixing well by vortex, and incubating at 60 °C for 10min. The AE of hybridized probe was not hydrolyzed under these conditions. The tubes were cooled on ice for over 1min, and chemiluminescence was measured for 2 s per tube with a luminometer (BPCL-2-TGC, Ultra Weak Luminescence Analyzer). For normalization of genomic DNA amount in each assay, we used A1a mouse repetitive HPA probe (5' GAACAGTGTATA TCAATGAGTTA CAAT- 3'; AE position is underlined). To check specificity of G-tail detection, non-denatured genomic DNA was treated with Exonuclease I (30 U/μg DNA) at 37° C for overnight, and heat inactivated at 80 °C for 20 min, before G-tails were assayed. Probes for AE-labeling of telomeres and detection of *Alu* sequences were supplied by Bioneer Inc. (Korea).

Telomere-TdT Assay

Labeling unprotected telomeres with cy3 conjugated deoxy-Uridine (Amersham) was performed essentially as described previously (1). Co-localization of the TdT signal with telomeres was performed by fixing TdT-labeled cells with 2% paraformaldehyde, washing 3 times in PBS, followed by incubation with anti-TRF1 antibody, and visualized with fluorescein conjugated secondary antibody. Images were taken using an Olympus Fluoview FV1000 confocal microscope.

Apoptosis analysis

Surface exposure of phosphatidylserine in apoptotic cells was measured using an AnnexinV/FITC and PI apoptosis detection kit (KeyGEN, Nanjing, China). The cells

were collected and resuspended in the binding buffer provided in the kit, then mixed with PI and FITC conjugated Annexin V. After incubation for 15 min, the cells were assessed via flow cytometric analysis.

SA-β-Gal assay

Cells treated with NiM and NiP were washed twice in PBS, fixed in 2% formaldehyde/0.2% glutaraldehyde for 5 min at room temperature, washed again in PBS, and incubated for 16h with β-Gal stain solution containing 1 mg/ml 5-bromo-4-chloro-3-indolyl-β-D-galactoside, 40 mM citric acid/sodium phosphate (pH 6), 5 mM potassium ferrocynide, 5 mM ferricyanide, 150 nM NaCl and 2 mM $MgCl_2$. Cells were viewed with a OLYMPUS BX-51 light microscope and photographed.

Immunoblotting analysis

Cells after treatment were washed with PBS, and lysed in 100 μL of lysis buffer (10 mM Tris-HCl, pH 7.4, 5 mM $MgCl_2$, 1 mM EDTA, 25 mM NaF, fresh 100 mM Na_3VO_4 and l mM dithiothreitol). Cell lysates were centrifuged for 10 min at 12000g. Concentrations of protein in the supernatant were determined by Bradford protein assay. Equal amounts of protein (40 μg) were resolved on 10% SDS-PAGE, and transferred electrophoretically to PVDF membrane. The membranes were blocked with nonfat dry milk (5%) in PBST (10 mM Tris-HCl, pH 7.4, 150 mM NaCl, 0.05% Tween-20), and then incubated with primary antibodies for 2 h at room temperature, washed three times with PBST, and then incubated with secondary antibody (HRP-conjugated) for 2h at room temperature. After washing the secondary antibody,

the bound antibody complex was detected using an ECL chemiluminescence reagent (Thermo). The primary antibodies used in this experiment were: mAb anti-TRF1 (Novus), mAb anti-TRF2 (Novus), pAb anti-POT1 (Sigma), mAb anti-γ-H2AX (Genscript), pAb anti-H2AX (Genscript), mAb anti-hTERT (Rockland), anti-p21 and anti-p16 (Thermo Fisher Scientific), β-actin (Sigma), and anti-myc-tag (Santa Cruz).

Inductively coupled plasma mass spectrometry (ICP-MS)

Cells treated with NiM and NiP were washed five times in PBS, and lysed with 500 μL of 5% SDS. Cell lysates were centrifuged for 20 min at 12000g. Concentrations of Ni in the supernatant were determined by ICP-MS.

1. Chen, Y., Qu, K., Zhao, C., Wu, L., Ren, J., Wang, J. and Qu, X. (2012) *Nat Commun*, **3**, 1074.
2. Tahara, H., Kusunoki, M., Yamanaka, Y., Matsumura, S. and Ide, T. (2005) *Nat Meth*, **2**, 829-831.

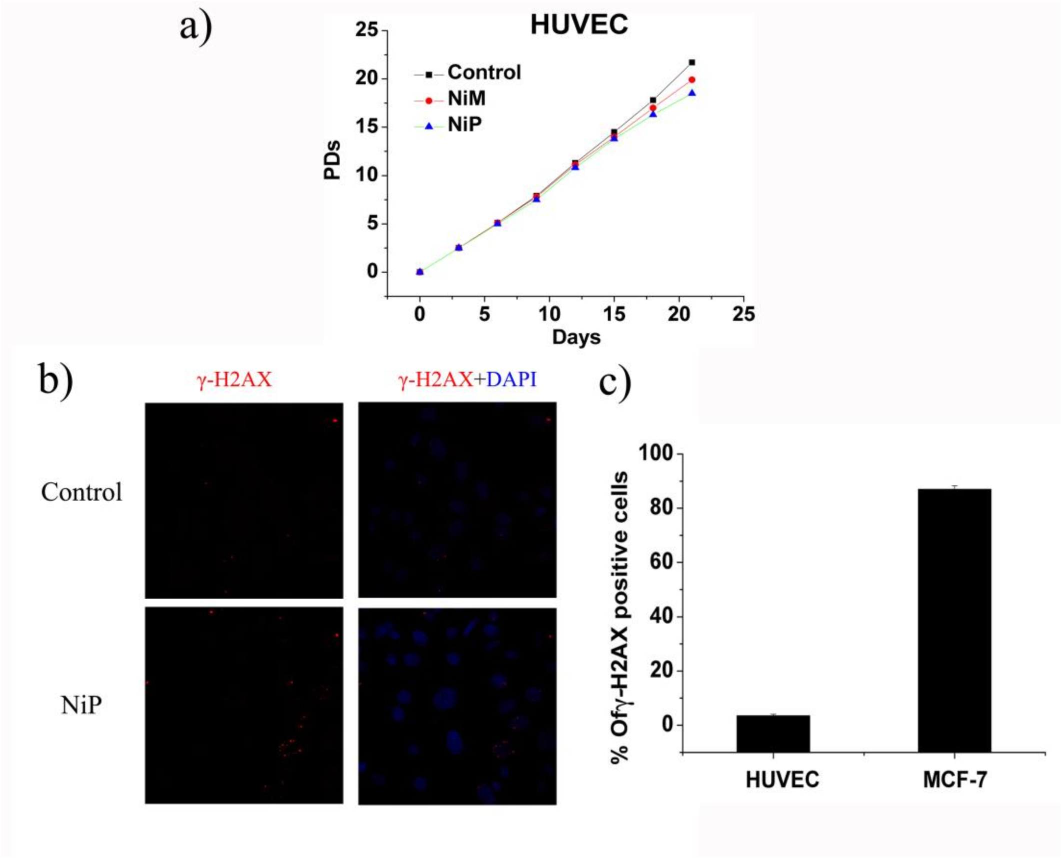

Figure S1 (a) Proliferation curves of HUVEC cells treated with NiM and NiP (15 μM). At the indicated times, cells were counted and the PDs were determined. (b) Representative immunofluorescence images of γ-H2AX and 53BP1 foci in HUVEC cells treated with NiP. (c) Percentage of cells containing γ-H2AX foci in HUVEC and MCF-7 cells treated with NiP for 24 h.

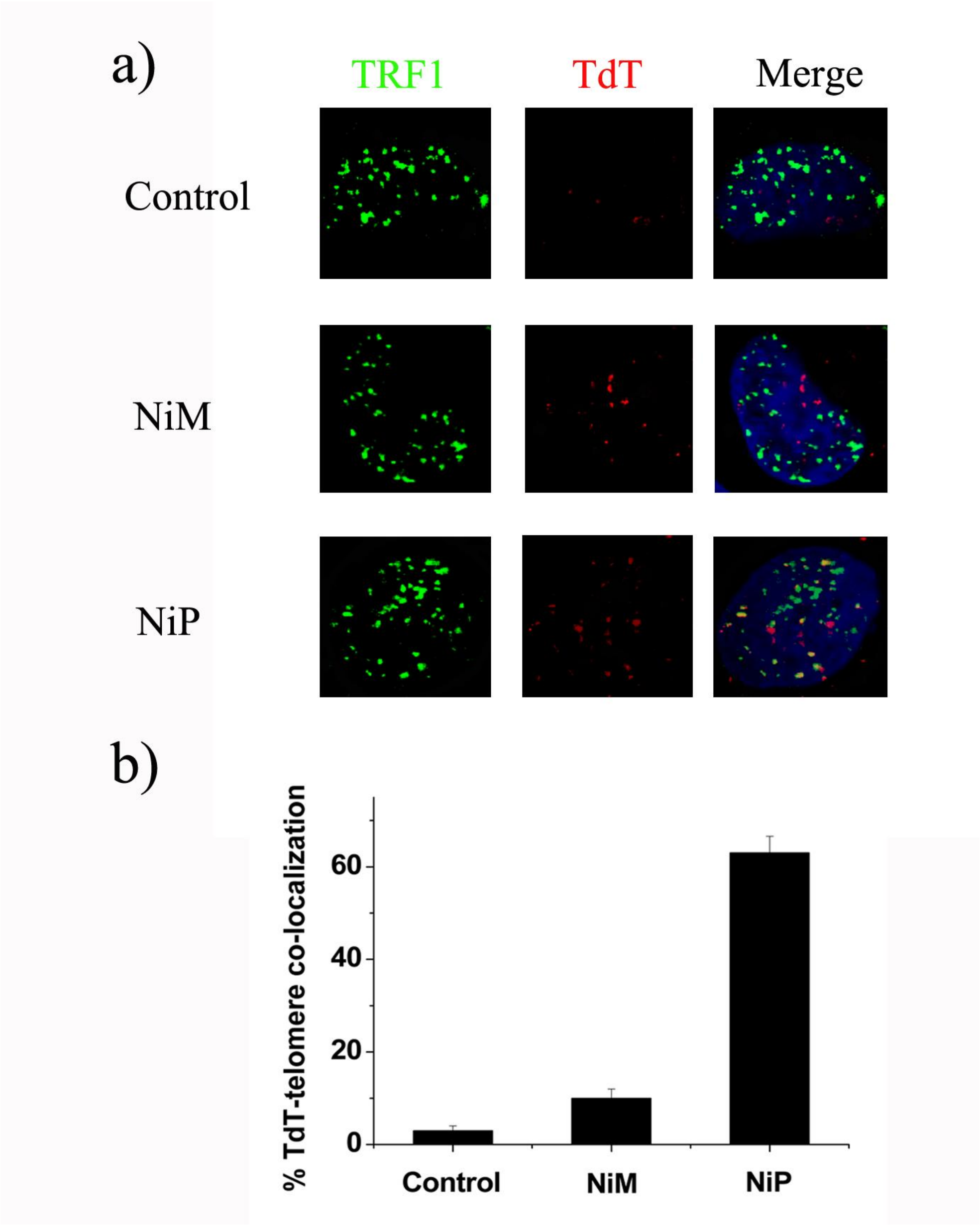

Figure S2 (a) NiP induced accessible telomere ends. TRF1 (*green*) were used to detect telomeres, while TdT-cy3 (*red*) was used as a marker of uncapped telomeres in MCF-7 cells treated with NiM and NiP. Merged signals were shown in the right. (b) Quantification of the percentage of TdT-cy3-positive cells in NiM and NiP. A minimum of 100 nuclei was scored, and error bars represented standard error of the derivation (SD).

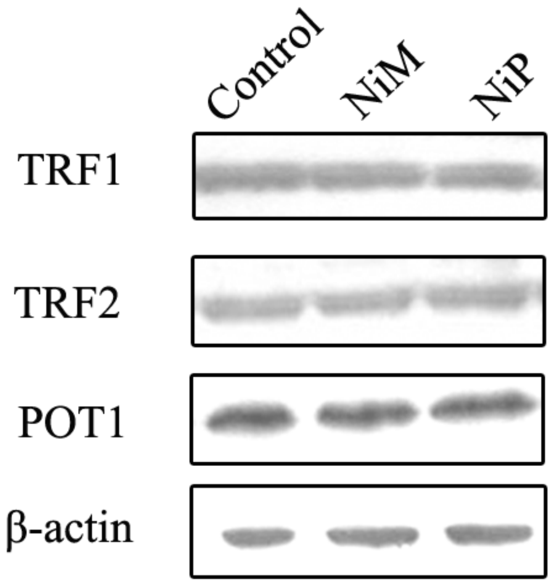

Figure S3 Expression of TRF1, TRF2 and POT1 in MCF-7 cells treated with NiM and NiP. β-actin was used as loading control.

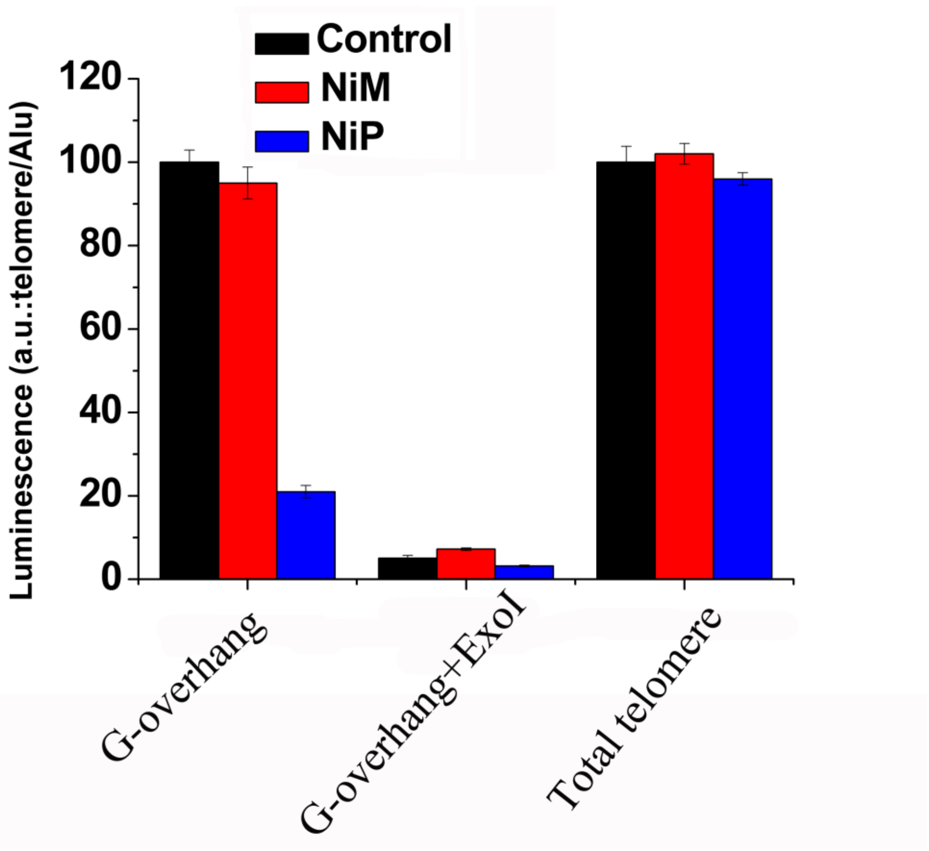

Figure S4 Hybridization protection assay (HPA) was performed on genomic DNA isolated from MCF-7 cells treated with NiM and NiP to assess the length of G overhang and total telomere length. *ExoI* nuclease digestion was used to assess integrity of the 3′ overhang. Luminescence intensity in arbitrary units (AU) was normalized against *Alu* probe. The mean of three independent experiments with comparable results was shown. Error bars indicated SD.

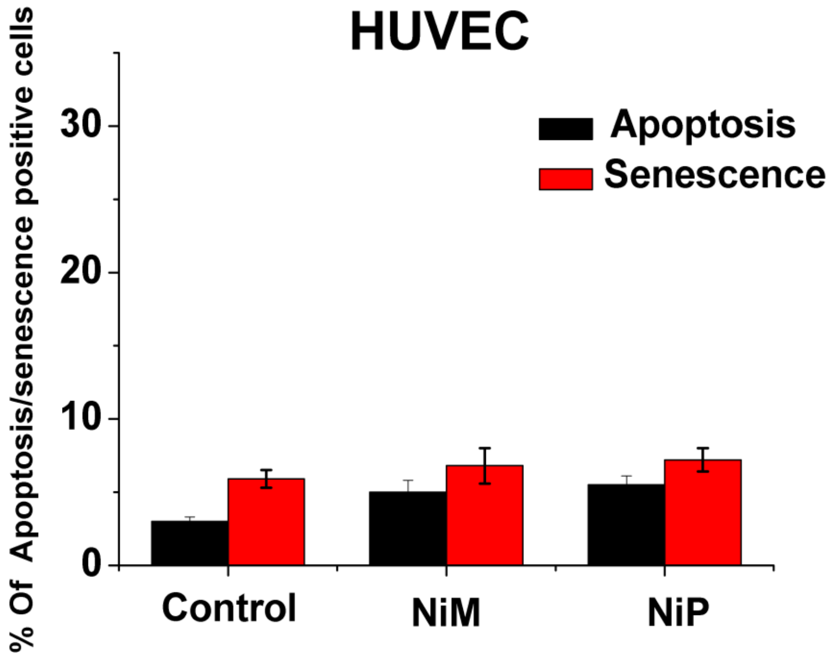

Figure S5 NiP did not induce apoptosis and senescence in HUVEC cells. Apoptotic cells were stained by PI and Annexin V-FITC, and measured by flow cytometry. Senescence cells were detected by assaying the SA-β-gal activity.
